# Disruption of Microbial Biofilms by an Extracellular Protein Isolated from Epibiotic Tropical Marine Strain of *Bacillus licheniformis*

**DOI:** 10.1371/journal.pone.0064501

**Published:** 2013-05-15

**Authors:** Devendra H. Dusane, Samir R. Damare, Yarlagadda V. Nancharaiah, N. Ramaiah, Vayalam P. Venugopalan, Ameeta Ravi Kumar, Smita S. Zinjarde

**Affiliations:** 1 Institute of Bioinformatics and Biotechnology, University of Pune, Pune, India; 2 Gene Laboratory, National Institute of Oceanography, Dona Paula, Goa, India; 3 Biofouling and Biofilm Process Section, Water and Steam Chemistry Division, BARC Facilities, Kalpakkam, India; University Paris South, France

## Abstract

**Background:**

Marine epibiotic bacteria produce bioactive compounds effective against microbial biofilms. The study examines antibiofilm ability of a protein obtained from a tropical marine strain of *Bacillus licheniformis* D1.

**Methodology/Principal Findings:**

*B. licheniformis* strain D1 isolated from the surface of green mussel, *Perna viridis* showed antimicrobial activity against pathogenic *Candida albicans* BH, *Pseudomonas aeruginosa* PAO1 and biofouling *Bacillus pumilus* TiO1 cultures. The antimicrobial activity was lost after treatment with trypsin and proteinase K. The protein was purified by ultrafiltration and size-exclusion chromatography. Sodium dodecyl sulfate-polyacrylamide gel electrophoresis (SDS-PAGE) and matrix assisted laser desorption/ionization-time of flight (MALDI-TOF) analysis revealed the antimicrobial agent to be a 14 kDa protein designated as BL-DZ1. The protein was stable at 75°C for 30 min and over a pH range of 3.0 to 11.0. The sequence alignment of the MALDI-fingerprint showed homology with the NCBI entry for a hypothetical protein (BL00275) derived from *B. licheniformis* ATCC 14580 with the accession number gi52082584. The protein showed minimum inhibitory concentration (MIC) value of 1.6 µg/ml against *C. albicans*. Against both *P. aeruginosa* and *B. pumilus* the MIC was 3.12 µg/ml. The protein inhibited microbial growth, decreased biofilm formation and dispersed pre-formed biofilms of the representative cultures in polystyrene microtiter plates and on glass surfaces.

**Conclusion/Significance:**

We isolated a protein from a tropical marine strain of *B. licheniformis*, assigned a function to the hypothetical protein entry in the NCBI database and described its application as a potential antibiofilm agent.

## Introduction

Biofilms are microbial communities that grow on different biotic and abiotic surfaces. Biofilms are often detrimental in nature and are particularly significant in the medical and industrial fields [1]. A variety of antimicrobial agents have been used to control biofilms. However, factors like lower efficacy and increased resistance of the biofilms towards these antimicrobial agents limit their effective applications [2]. This has led to a search for natural products as alternative antibiofilm agents.

Marine ecosystems are potential repertoires of bioactive compounds [3]. Some of these natural products are reported to be effective in controlling detrimental biofilms [4], [5]. Marine epibiotic bacteria live in a highly competitive environment where they encounter a limitation for space. In order to colonize a surface and to ward-off competition, they often produce bioactive compounds and thus play an important role in marine ecology [6], [7]. Antimicrobial compounds have been isolated from marine sponges [8], algae [9], ascidians [10], sea grasses [11], sea stars [12] and sea pansies [13]. There is increasing evidence that the bacteria associated with such marine biological forms are responsible for the production of antimicrobials isolated from them [4], [5], [14], [15].

Although bioactive compounds from marine microorganisms have been exploited for decades; their applications in treating detrimental biofilms is an area that is relatively less-explored [16]. In this regard, bacteria such as *Pseudoalteromonas tunicata*, *Brevibacterium casei*, *Vibrio sp.* and *Serratia marcescens* are reported to produce biofilm disrupting agents [17], [18], [19], [20], [21]. In the present investigation we (i) purified a protein (designated BL-DZ1) from the marine epibiotic *B. licheniformis* strain D1 (ii) assigned a function to the NCBI entry and (iii) showed the effectiveness of this protein in dispersing representative bacterial and fungal biofilms.

## Materials and Methods

### Microorganisms, growth conditions and antimicrobial compound production

A tropical marine strain of *B. licheniformis* D1 was used in the study [22]. The bacterium was grown in Luria Bertani (LB) broth containing tryptone: 10.0; yeast extract: 5.0; sodium chloride: 1.0 g/l of distilled water, pH 7.0 at 30°C with shaking for 48 h. Samples were intermittently withdrawn and growth was monitored at 600 nm. The culture broth was centrifuged at 7000× *g* for 10 min and the supernatant was filter- sterilized by passing through 0.22 µ filter (Millipore, USA). The cell-free supernatant (CFS) thus obtained was assessed for antimicrobial activity against *Candida albicans* BH, *Pseudomonas aeruginosa* PAO1 (medically important microorganisms) and *Bacillus pumilus* TiO1 (a biofouling bacterium) by using the agar well-diffusion method [21]. *C. albicans* was grown in YPD medium (yeast extract: 10.0; peptone: 20.0; dextrose: 20.0 g/l of distilled water). *P. aeruginosa* and *B. pumilus* were grown in LB broth.

### Purification of *B. licheniformis* antimicrobial protein (BL-DZ1)

To determine the type of antimicrobial compound, bacterial cell free supernatant (1 ml) was treated with proteinase K (10 mg/ml; Sigma-Aldrich, USA) and trypsin (10 mg/ml; Sigma-Aldrich, USA) at 30°C for 1 h. The antimicrobial activity of the protein/peptide in the supernatant was determined against the test cultures after inactivating the enzyme by incubating at 100°C for 5 min. The heat treatment step had no effect on the antimicrobial activity of the protein/peptide. Treatment with proteinase K and trypsin resulted in loss of antimicrobial activity, suggesting the antimicrobial compound to be a protein or a peptide. The protein was isolated by cultivating *B. licheniformis* D1 cells in 1000 ml of LB broth (30°C, 120 rpm, 36 h). The cell-free supernatants (0.22 µ filtered) were concentrated in an Amicon ultrafiltration system (Millipore, USA) using a 3 kDa cut-off membrane. The retentate that displayed antimicrobial activity was subjected to size-exclusion chromatography (Superdex 200 column, Amersham Biosciences, Upssala, Sweden). The bioactive protein was eluted (with 0.2 M NaCl in 100 mM Tris buffer, pH 7.5 using a flow rate of 0.5 ml/min) and the fractions were tested for antimicrobial activity. The elution volumes of the bioactive protein and standard pure proteins (BSA, chicken egg albumin, carbonic anhydrase, α-lacto albumin) for the column at the same flow rate were also determined. Calibration curves were obtained and used to determine the molecular mass of the bioactive protein.

At each step of purification, the antimicrobial activity and protein content [23] were determined. To evaluate the antimicrobial activity during the purification steps, arbitrary units (reciprocal of maximum dilution showing zone of inhibition) were determined. All experiments were carried out in triplicates using two biological replicates and representative data are presented here.

### SDS-PAGE and in-gel-digestion with trypsin

The protein purity and molecular mass was ascertained using SDS-PAGE [24]. Electrophoresis was carried out in 15% polyacrylamide gels at a constant voltage (60V) and the proteins were detected by silver staining [25]. “In-gel-digestion” with trypsin was performed in SDS-PAGE gels that were stained with Coomassie brilliant blue G-250. Protein bands were excised from the gel and rinsed three times for 10 min with water (HPLC grade, Merck Darmstadt Germany). Reduction was performed with 0.1 M Tris (pH 8.5) containing 0.01 M ethylenediaminetetraacetic acid, 6 M guanidine HCl and 25 mM dithiothreitol for 30 min at 37°C. The proteins were subsequently alkylated with 125 mM iodoacetamide in dark for an additional period of 1 h at 37°C. Gel pieces were equilibrated twice with 100 ml of 50 mM ammonium bicarbonate (NH_4_HCO_3_, pH 7.8) for 10 min, shrunk with 100 ml of acetonitrile, rehydrated with 100 ml of 50 mM NH_4_HCO_3_ (pH 7.8) and finally shrunk with acetonitrile. After air-drying, gel pieces were re-swollen in a digestion buffer, containing 20 ml of 50 mM NH_4_HCO_3_, and 0.05 mg of trypsin (Sigma-Aldrich, USA) at 37°C for 16 h. Peptides were extracted by subsequent incubation with 50 mM NH_4_HCO_3_, 0.1% trifluoroacetic acid for 20 min at room temperature and finally with 0.1% TFA: acetonitrile (2:3, v/v). The pellet was dissolved in 10 ml of 0.1% TFA.

### MALDI-TOF analysis

The digested protein was applied onto the target plates and subjected to MALDI-TOF analysis (Applied Biosystems, USA). The matrix used was α-hydro-cyano-cinnammic acid (CHCA). Proteins were identified from MALDI-fingerprint data using a locally installed MASCOT. The sequence alignment was carried out by using the BLAST programme (http://www.ncbi.nlm.nih.gov).

### Effect of proteolytic enzymes, pH and temperature on antimicrobial activity of the pure protein

The purified protein was treated with proteinase K (10 mg/ml; Sigma-Aldrich, USA) and trypsin (10 mg/ml; Sigma-Aldrich, USA). Thermal stability of the protein with respect to antimicrobial activity was checked by incubating the protein at 30, 40, 50, 60, 70, 80 or 100°C for 30 min. The influence of pH on the antimicrobial activity was examined by varying the pH at 3.0, 5.0, 7.0, 9.0 or 11.0 and incubating for 2 h at 30°C [26]. The residual antimicrobial activity against the test cultures was determined by using the agar well-diffusion assay.

### Determination of minimum inhibitory concentration (MIC)

MIC of the purified protein was determined by the broth micro-dilution assay according to Clinical Laboratory Standards Institute (CLSI) guidelines. The protein (100 μg/ml) was diluted with Mueller-Hilton broth (HiMedia, India) in 96-well microtiter plates (100 μl). To each of these wells, 100 μl of test cultures (*C. albicans, P. aeruginosa* or *B. pumilus*) containing 5×10^5^ colony forming units per ml were added. After 24 h of incubation at 37°C (*C. albicans* and *P. aeruginosa*) or 30°C (*B. pumilus*) as otherwise stated, the wells were inspected for microbial growth and the MIC was defined as the lowest concentration that inhibited growth of the test cultures. Standard antimicrobial agents, fluconazole (Sigma, India), tetracycline (Sigma-Aldrich, India) and nalidixic acid (Fluka, India) were used for comparison. All experiments were carried out in triplicates with two biological replicates and representative data is presented here.

### Inhibition or disruption of biofilms in 96 well polystyrene microtiter plates

In order to determine the ability of purified protein to inhibit biofilms, the test cultures were allowed to grow in 96 well polystyrene microtiter plates in the presence of protein (0.1–100 µg/ml). The plates were incubated for 24 h after which the medium was aspirated. The wells were gently rinsed with phosphate buffer (50 mM, pH 7.0), air-dried, and the biofilms were quantified using the crystal violet assay [27]. Microtiter plate wells containing bacterial or fungal cells without antimicrobial protein were used as controls during experimentation. The results were expressed in terms of percent biofilm formed in presence of protein compared to untreated wells (indicating 100% biofilm coverage). Fluconazole, tetracycline and nalidixic acid (2.5–2500 µg/ml) were used as standard antimicrobial agents for comparison. The data related to these experiments are depicted as average values of triplicate observations and error bars indicate standard deviation.

In order to determine the ability of the purified protein to disrupt pre-formed biofilms, the test cultures were formed in microtiter plate wells for 24 h. The pre-formed biofilms were then treated with the purified protein (0.1–100 µg/ml) for 24 h and the residual biofilm was estimated using crystal violet assay.

### CLSM analysis

Cells of the test cultures were co-incubated with the protein (BL-DZ1) or the antibiotics at respective MIC concentrations. The biofilms were allowed to form on pre-sterilized microscopic glass surfaces submerged in 20 ml of appropriate growth media in sterile Petri dishes for 24 h on a rocker. After incubation period, the slides were removed, rinsed twice with sterile phosphate buffer (50 mM, pH 7.0) to remove the planktonic cells and the biofilms were stained with *BacLight* Live/Dead stain (Molecular Probes, Eugene). The cell viability was assessed by using a confocal laser scanning microscope (CLSM, Leica, Germany). Triplicate experiments were performed and representative images are presented here.

For studies on the disruption of pre-established biofilms, aliquots (200 µl) of *C. albicans*, *P. aeruginosa* or *B. pumilus* broth cultures (grown for 12 h) were inoculated in sterile Petri plates containing 20 ml of appropriate growth media. Pre-sterilized microscopic glass slides were immersed in these media. The Petri plates were incubated at respective temperatures for 24 h on a rocker. After incubation, the slides were placed in fresh medium containing protein or antimicrobial agents at respective MIC concentrations. After further incubation for 24 h, the slides were removed, rinsed twice with phosphate buffer and stained with Live/Dead *BacLight* viability stain.

### Microscopy and image analysis

A confocal laser scanning microscope (TCS SP2 AOBS) equipped with DM IRE 2-inverted microscope (Leica Microsystems, Germany) was used to image the biofilms. The microscopy and image analysis was carried out as described earlier [21]. Control experiments without antimicrobial agents were considered to depict 100% coverage and the percent disruption for test samples was appropriately calculated.

### Scanning electron microscope (SEM) analysis

A representative strain of *P. aeruginosa* PAO1 was used for SEM experiments. Biofilms of *P. aeruginosa* were allowed to form on pre-sterilized glass surfaces placed in different wells of a 24 well microtiter plate (Tarsons, India) containing 3 ml LB broth. Effect of co-incubation with the protein as well as the dispersion of pre-established biofilms was monitored by SEM as described earlier [21]. Biofilms without protein treatment were used as controls.

### Statistical analysis

The effect of antimicrobial agents on fungal and bacterial biofilm formation was estimated by one way analysis of variance (ANOVA) using Origin 6.0 software. The observations were evaluated statistically by using the Students *t*-test and treatments were considered significant when P≤0.05.

## Results

In our earlier investigation on cross-species induction of bioactive compounds, few tropical marine epibiotic bacterial strains were isolated [22]. Among these, a strain of *B. licheniformis* D1 obtained from the surface of the green mussel, *P. viridis* displayed excellent antimicrobial activity. This strain was used in the study and we report the purification, characterization of an antimicrobial protein from this marine bacterium. In addition, we also demonstrate the application of the purified protein against *P. aeruginosa*, *B. pumilus* and *C. albicans* biofilms.

### Growth characteristics and antimicrobial activity displayed by *B. licheniformis* D1

**Figure 1:**
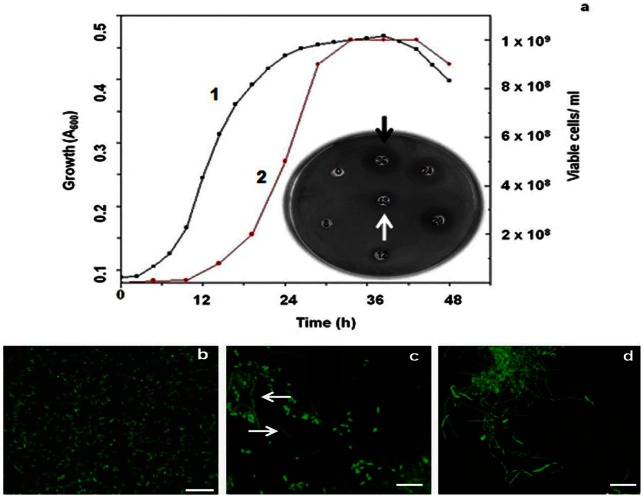
Growth characteristics of ***Bacillus licheniformis*** **in LB broth.** Line 1 depicts growth estimated as A_600_ and line 2 depicts viable cells/ml. Inset is a representative plate showing antimicrobial activity over a period of time with 10 µl of the cell free supernatants. Morphology of *B. licheniformis* D1 grown in LB after (b) 18 h (c) 24 h (d) 96 h. Bar represents 10 µm.

*B. licheniformis* D1 growth in terms of absorbance at 600 nm (Figure 1a, line 1) as well as viable cell counts (Figure 1a, line 2) were determined. An initial lag phase (0 to 8 h), an exponential phase (8 to 24 h) and a subsequent stationary phase was observed. Samples were withdrawn at intermittent time intervals and evaluated for antimicrobial activity. A representative image (Figure 1a inset) shows time-dependent production of antimicrobial compound against *P*. *aeruginosa*. The antimicrobial activity was evident after 12 h of incubation and a maximum zone of inhibition was seen at 36 h (black arrow). Thereafter, decrease in the bioactivity (white arrow) takes place. Morphological variations were observed during the growth phases. Short rods were predominant until 18 h (Figure 1b); these began to differentiate into long filaments after 24 h of growth (Figure 1c; white arrows). On further incubation (after 96 h), the bacterial cells mainly existed in filamentous forms (Figure 1d).

### Purification and characterization of the antimicrobial compound

**Figure 2:**
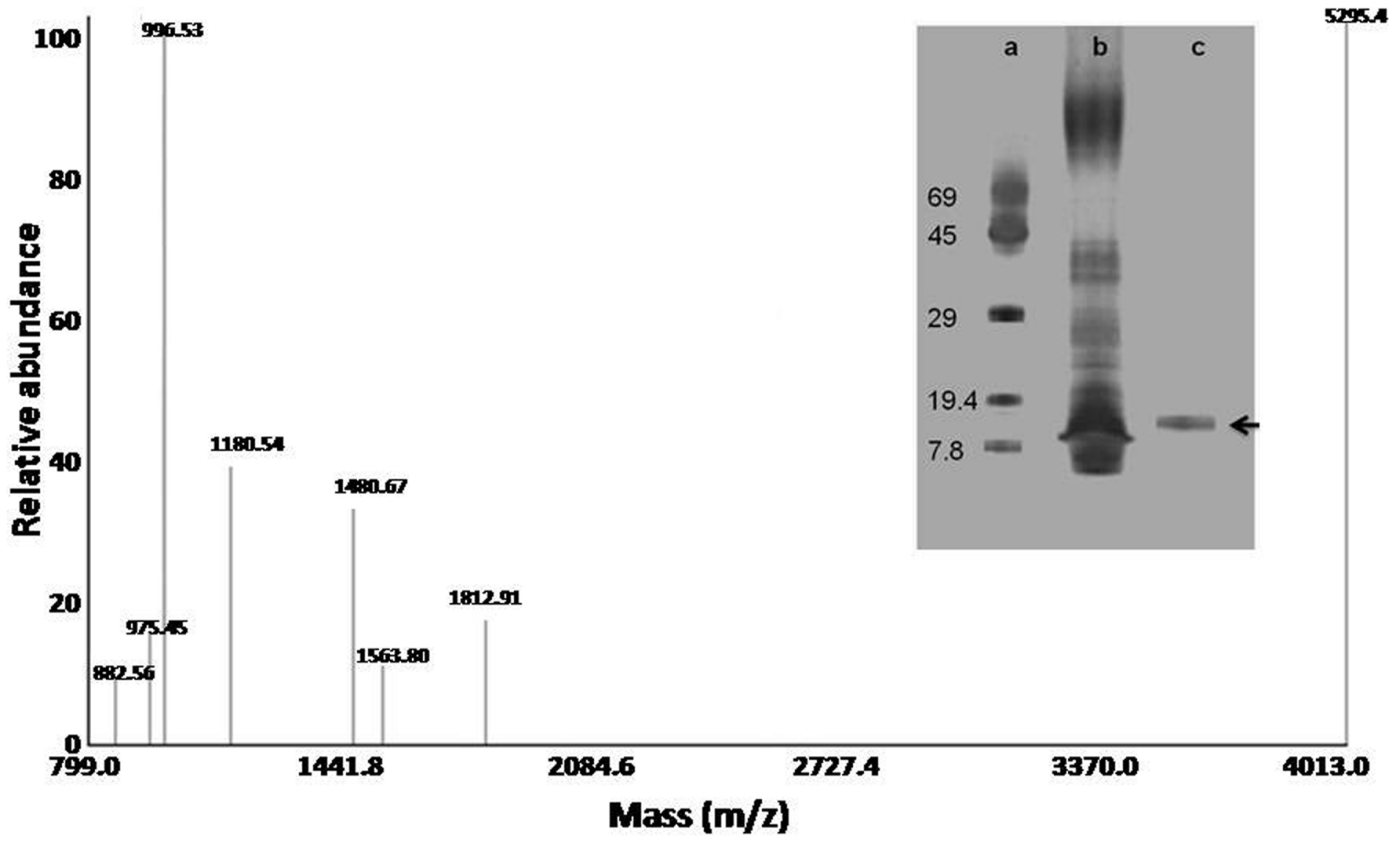
MALDI-TOF analysis of the tryptic digest fingerprint predicting the peptide mass to be 14 kDa. Inset is a representative SDS-PAGE profile of *B. licheniformis* proteins. Lane a: molecular weight markers; Lane b: 3 kDa retentate; Lane c: purified protein after size-exclusion chromatography.

After ultrafiltration of the cell-free supernatants, the retentate displayed antimicrobial activity. The retentate was subjected to size-exclusion chromatography; fractions were collected and re-analyzed for antimicrobial activity. A fraction showing maximum zone of inhibition was further characterized. The molecular mass was determined by three methods (i) size-exclusion chromatography (ii) SDS-PAGE and (iii) MALDI-TOF analysis of the tryptic digest fingerprints. From the plots of log molecular mass of standard proteins (BSA, chicken egg albumin, carbonic anhydrase, α-lacto albumin) versus elution volumes, the mass of the antimicrobial protein was estimated to be 14 kDa. The molecular mass was also determined by SDS-PAGE (Figure 2 inset). In this figure, lane ‘a’ depicts molecular mass markers, the retentate showed presence of several bands (lane ‘b’). The fraction showing the highest activity displayed a single protein band (lane ‘c’, black arrow). This data also indicated that the molecular mass of purified protein was 14 kDa. By following the aforementioned protocol, the antimicrobial protein BL-DZ1 was purified to homogeneity with a 47.05 fold purification (Table 1). The protein was excised, subjected to in-gel digestion, and analyzed by MALDI-TOF (Figure 2). MALDI-TOF analysis of the tryptic digest fingerprint was compared with the NCBI database. The fingerprint matched with an NCBI entry, for a hypothetical protein (BL00275 with an accession number gi52082584) from *B. licheniformis* ATCC 14580. The protein is reported to have a molecular mass of 14 kDa (Figure S1). The purified protein was stable at 75°C for 30 min and in the pH range between 3.0–11.0. The protein was however sensitive to the enzymes trypsin and proteinase K.

**Table 1:** Purification of the antimicrobial protein BL-DZ1 from *Bacillus licheniformis* D1.

Purification steps	Volume (ml)	Total protein (mg)	Total activity (AU)	Specific activity (AU/mg)	Fold Purification
Cell free supernatant	1000	128.61	64	0.5	1
Ultrafiltration (3kDa)	60	19.05	128	6.7	13.4
Gel filtration (Superdex 200)	10	5.44	128	23.5	47.05

AU: arbitrary unit is defined as the reciprocal of maximum dilution showing zone of inhibition.

**Table 2:** Quantitative data on biofilm disruption obtained from confocal laser scanning microscopic image analysis (N =  20; using *ImageJ* software).

Test cultures / conditions	Inhibition (%)							
	Protein		Tetracycline		Nalidixic acid		Fluconazole	
	½ MIC	MIC	½ MIC	MIC	½ MIC	MIC	½ MIC	MIC
*C. albicans* (CI)	89.0±2.1	96.8±1.1	ND	ND	ND	ND	63.5±3.6	85.0±1.1
*C. albicans* (PF)	65.5±1.5	83.4±4.2	ND	ND	ND	ND	55.5±5.5	59.4±3.2
*P. aeruginosa* (CI)	85.8±3.2	92.2±2.4	73.0±5.1	80.0±4.1	28.0±4.4	41.8±3.1	ND	ND
*P. aeruginosa* (PF)	76.1±1.1	88.9±2.5	55.7±1.1	60.8±1.9	11.6±2.4	38.9±1.5	ND	ND
*B. pumilus* (CI)	81.0±5.0	90.6±2.8	63.6±3.0	79.2±4.4	15.0±2.5	36.1±1.8	ND	ND
*B. pumilus* (PF)	79.4±2.6	90.1±2.1	44.5±2.2	68.5±5.4	10.1±4.8	30.5±5.0	ND	ND

CI  =  co-incubation; PF =  pre-formed biofilm disruption; ND  =  Not Determined

### Determination of minimum inhibitory concentration (MIC)

The antimicrobial protein had MIC value of 1.60 µg/ml (0.114 nM) against *C. albicans*. Against *P. aeruginosa* and *B. pumilus*, the MIC was 3.12 µg/ml (0.228 nM). Fluconazole displayed MIC value of 160 µg/ml (522 nM) against *C. albicans*. Tetracycline showed the MIC values of 40 and 80 µg/ml (90 and 180 nM) against *P. aeruginosa* and *B. pumilus*, respectively. With nalidixic acid, the MIC values were 1250 µg/ml and 2500 µg/ml (4910 and 9800 nM) for *P. aeruginosa* and *B. pumilus*, respectively. The protein was more effective against the test cultures at low concentrations when compared to commercially available antimicrobial agents (fluconazole, tetracycline and nalidixic acid).

### Inhibition of biofilm growth and disruption of pre-formed biofilms

**Figure 3:**
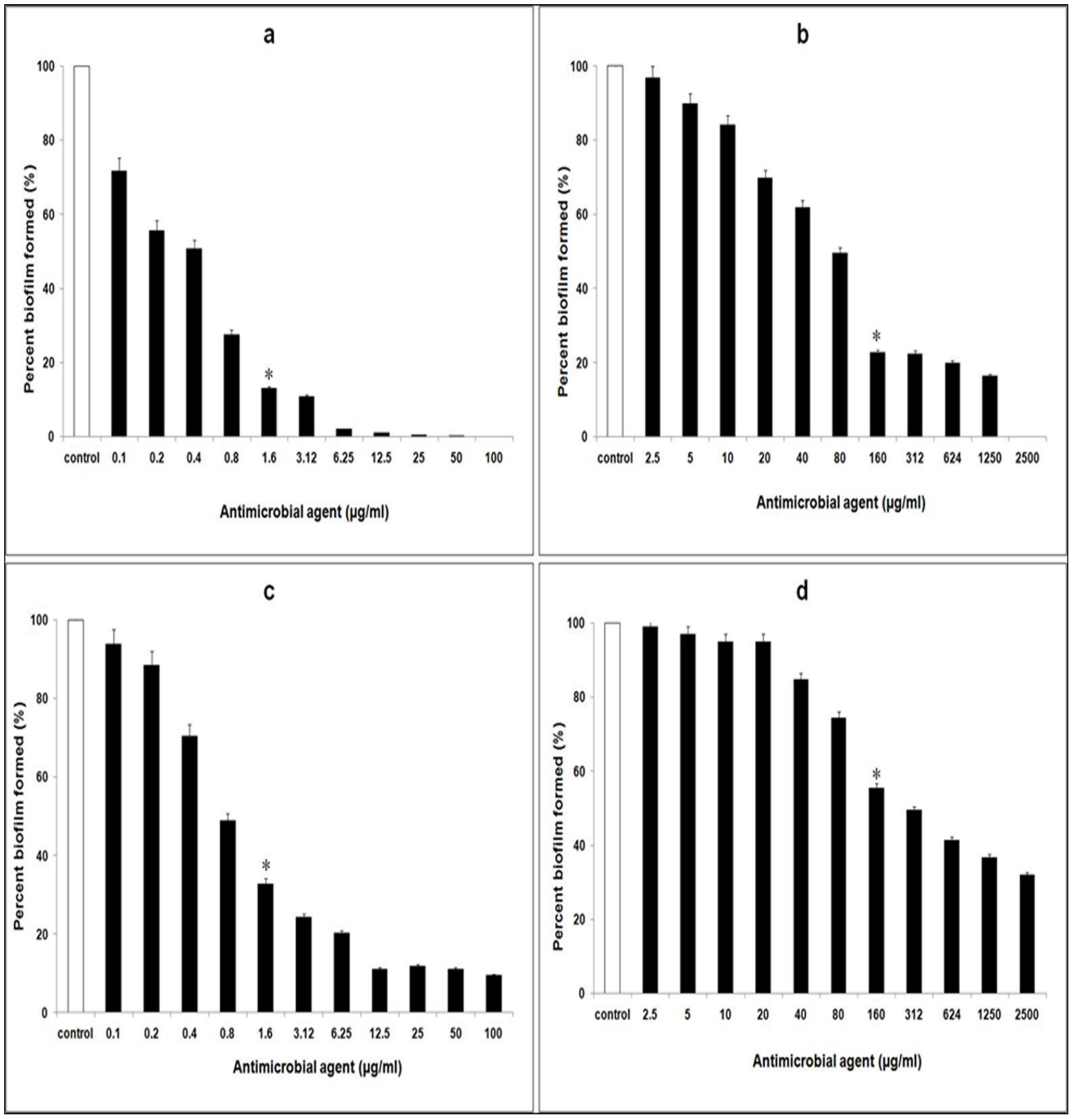
Inhibition of *C.* *albicans* biofilms after co-incubation with (a) *B. licheniformis* BL-DZ1 protein (b) fluconazole. Disruption of pre-formed biofilms by (c) *B. licheniformis* BL-DZ1 protein (d) fluconazole [* =  MIC value].

Compared to control biofilms (depicting 100% coverage), in the presence of the protein BL-DZ1 at MIC concentrations, a considerable reduction in biofilm formation was observed. In the presence of 1.60 µg/ml of the antimicrobial protein, biofilm formation by *C. albicans* decreased by 87.0% (P<0.01; Figure 3a). With fluconazole (160 µg/ml), the decrease was up to 77.2% (Figure 3b). Pre-formed biofilms of *C. albicans* were dispersed up to 67.2% by 1.6 µg/ml of the protein BL-DZ1 (P<0.05; Figure 3c) as compared to fluconazole (Figure 3d) that showed 44.5% reduction at 160 µg/ml.

**Figure 4:**
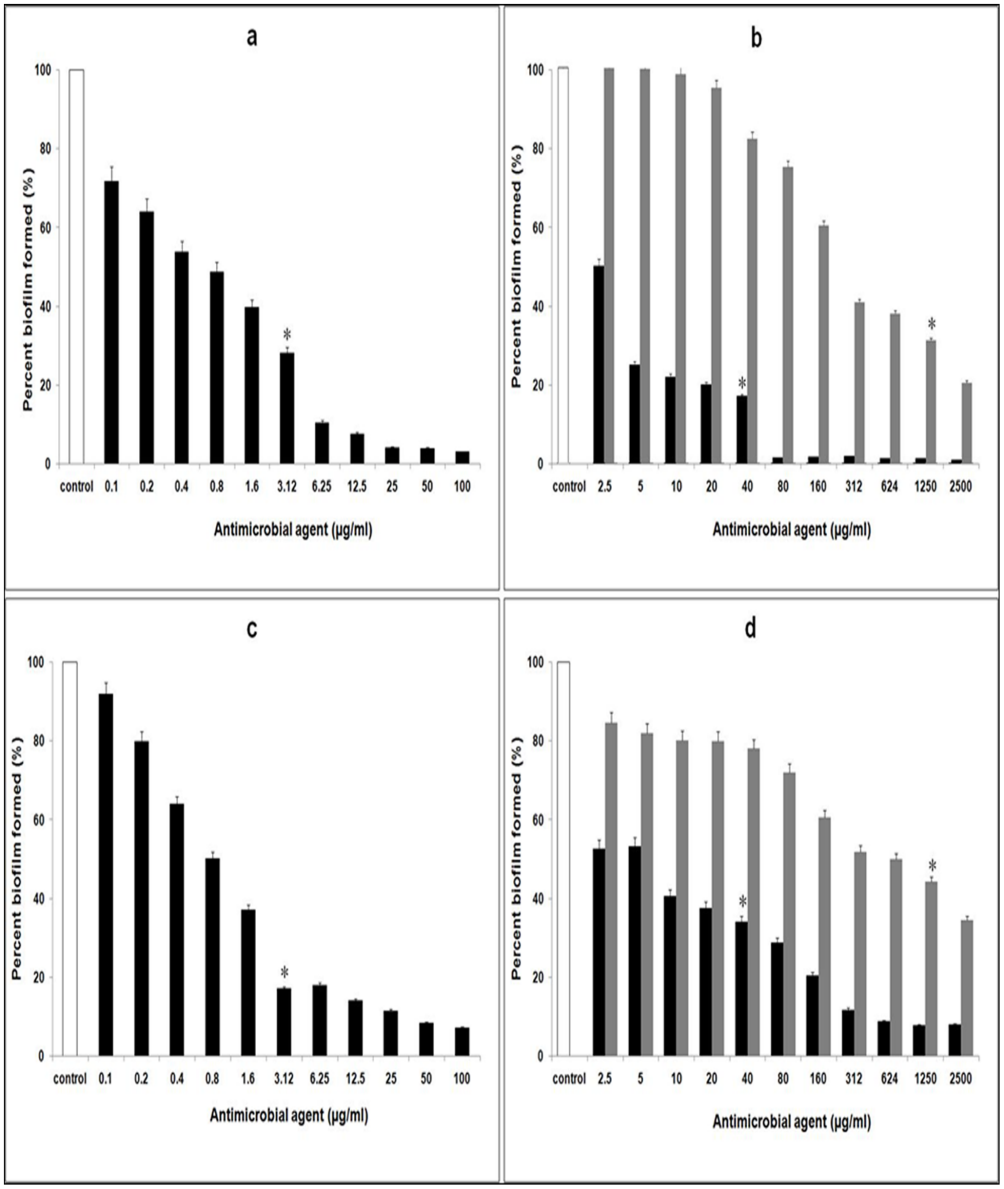
Inhibition of *P.* *aeruginosa* biofilms after co-incubation with (a) *B. licheniformis* BL-DZ1 protein (b) nalidixic acid (grey bars) and tetracycline (black bars). Disruption of pre-formed biofilms by (c) *B. licheniformis* BL-DZ1 protein (d) nalidixic acid (grey bars) and tetracycline (black bars) [* =  MIC value].

Similarly, the biofilm growth of *P. aeruginosa* was inhibited upto 71.79% with protein BL-DZ1 on co-incubation (Figure 4a). With tetracycline and nalidixic acid at MIC concentrations, biofilm growth was reduced by 82.9 and 68.8% (Figure 4b; black and grey bars, respectively). Pre-formed biofilms of *P. aeruginosa* when treated with MIC concentrations of protein BL-DZ1 showed 82.5% disruption (P =  0.01; Figure 4c). With tetracycline and nalidixic acid, this decrease was 65.8% and 55.8%, respectively (Figure 4d; black and grey bars, respectively).

**Figure 5:**
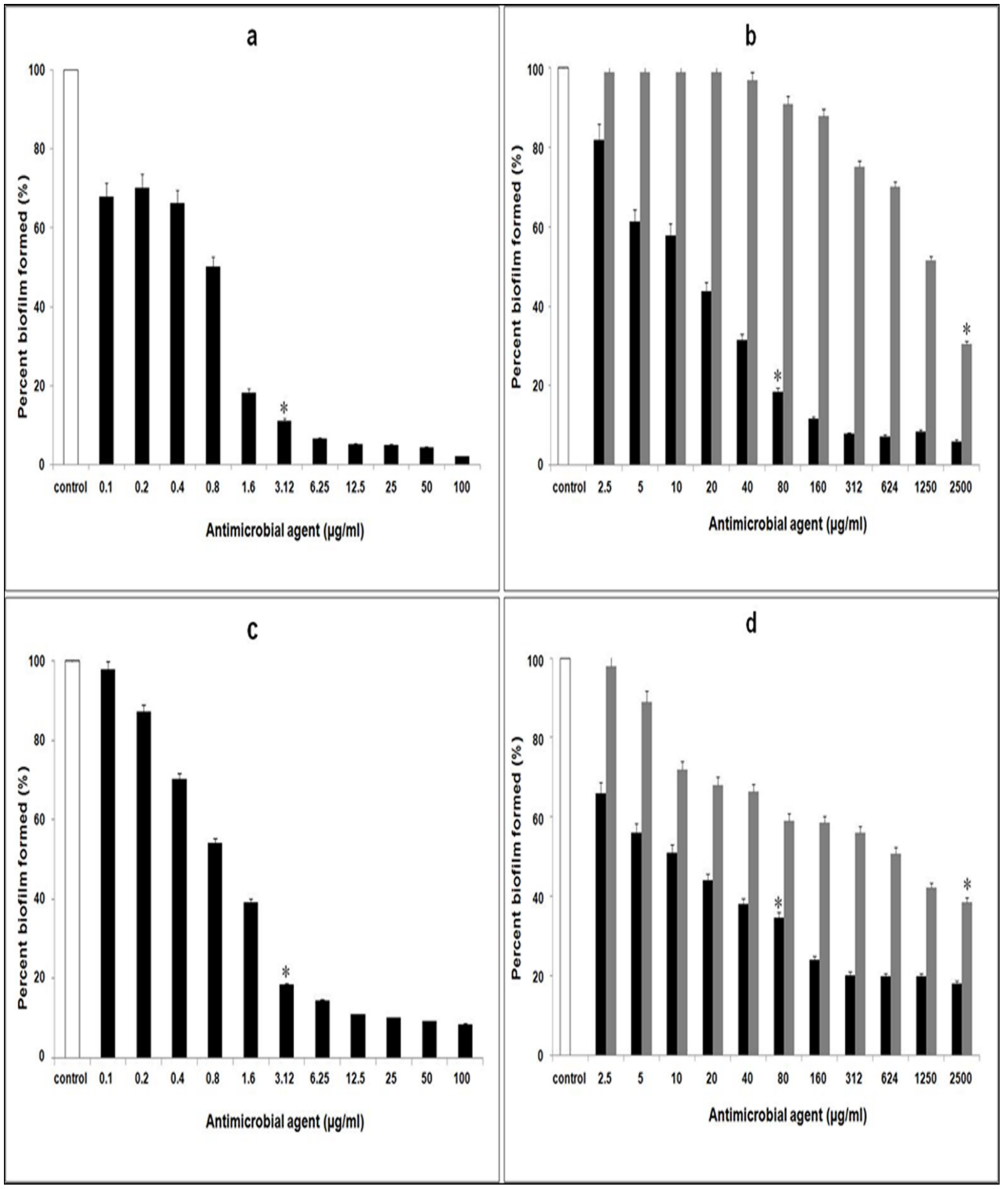
Inhibition of *B.* *pumilus* biofilms after co-incubation with (a) *B. licheniformis* BL-DZ1 protein (b) nalidixic acid (grey bars) and tetracycline (black bars). Disruption of pre-formed biofilms by (c) *B. licheniformis* BL-DZ1 protein (d) nalidixic acid (grey bars) and tetracycline (black bars) [* =  MIC value].

Biofilm formation by the biofouling bacterium, *B. pumilus* was also significantly inhibited (88.9%; P<0.01) by the protein BL-DZ1 (Figure 5a). With tetracycline and nalidixic acid this was 81.5 (P =  0.04) and 69.4% (P<0.05; Figure 5b; black and grey bars, respectively). Pre-formed biofilms of this bacterium were also effectively dispersed (81.5%) with the protein (Figure 5c). Lower values (65.5% and 61.5%) were observed with tetracycline and nalidixic acid (Figure 5d; black and grey bars, respectively).

Inhibition in both cases (after co-incubation and disruption of pre-formed biofilms) with all the test cultures was statistically significant with P<0.05 in case of the treated cells compared to the untreated controls.

### CLSM analysis of biofilm inhibition and disruption of pre-formed biofilms

**Figure 6:**
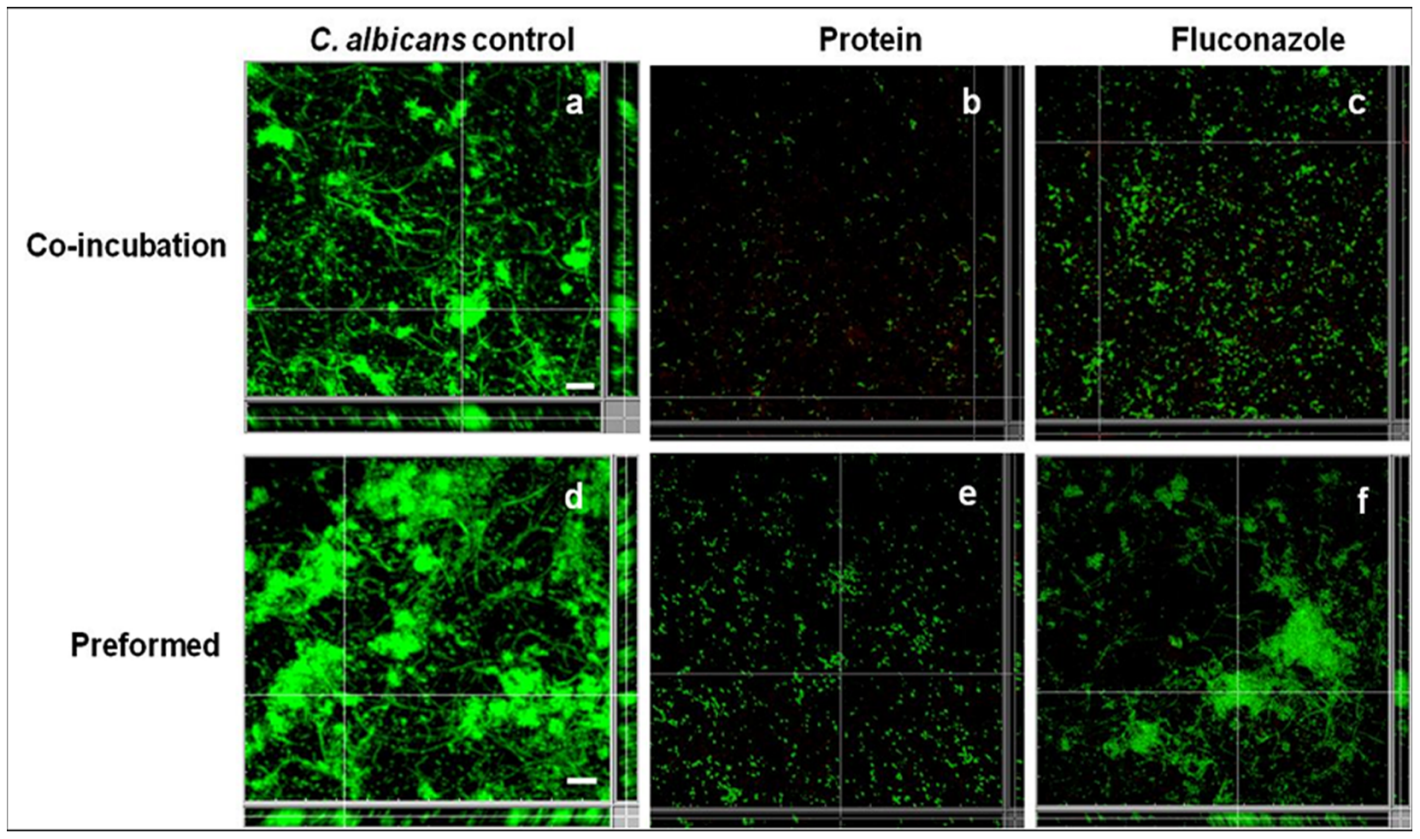
CLSM analysis of *C.* *albicans* biofilms (a) control. After co-incubation with (b) protein BL-DZ1 (c) fluconazole. Disruption of pre-formed biofilms (d) control, after treatment with (e) protein BL-DZ1 (f) fluconazole. Bar indicates 20 µm scale.

Biofilms of *C. albicans*, *P. aeruginosa* and *B. pumilus* were formed on glass slides in absence or presence of antimicrobial agents. A representative image of the control biofilms produced by *C. albicans* on glass surfaces is shown in Figure 6a. Protein BL-DZ1 mediated a significant (96.8%; P = 0.02) decrease in biofilm formation (Figure 6b) compared to a value of 85.0% observed with fluconazole (Figure 6c). A summary of the results obtained with co-incubation and disruption of pre-formed biofilms of the three cultures at MIC and ½ MIC values are depicted in Table 2.

Compared to control pre-formed biofilms of *C. albicans* (Figure 6d), the antimicrobial protein, mediated a disruption of 83.4% (P<0.01; Figure 6e). With fluconazole at MIC concentration the disruption was lesser, 59.4% (Figure 6f).

**Figure 7:**
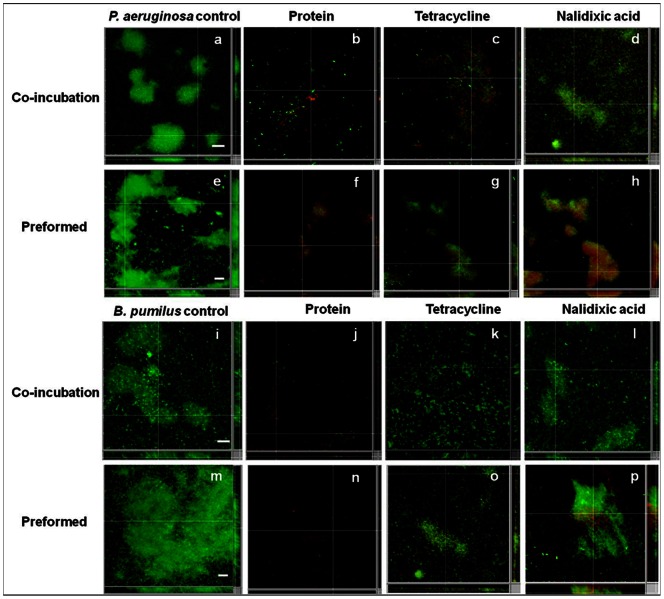
CLSM analysis of biofilms of *P.* *aeruginosa* (a) control. After co-incubation with (b) protein BL-DZ1 (c) tetracycline (d) nalidixic acid. Disruption of pre-formed biofilms (e) control, after treatment with (f) protein BL-DZ1 (g) tetracycline (h) nalidixic acid. CLSM analysis of biofilms of *B. pumilis* (i) control. After co-incubation with (j) protein BL-DZ1 (k) tetracycline (l) nalidixic acid. Disruption of pre-formed biofilms (m) control, after treatment with (n) protein BL-DZ1 (o) tetracycline (p) nalidixic acid. Bar indicates 20 µm scale.

Biofilm growth of *P. aeruginosa* (Figure 7a) was significantly inhibited (92.2%) after treatment with the antimicrobial protein (Figure 7b). Tetracycline and nalidixic acid were less effective in inhibiting the biofilms with values of 80.0% (Figure 7c) and 41.8% (Figure 7d), respectively. Compared to the untreated pre-formed biofilms of *P. aeruginosa* (Figure 7e), the antimicrobial protein significantly (88.9%) disrupted test biofilms (Figure 7f). With tetracycline and nalidixic acid, the disruption was less with values of 60.5% (Figure 7g) and 38.9% (Figure 7h), respectively.

Control biofilms of *B. pumilus* (Figure 7i) were inhibited with the antimicrobial protein to 90.6% (P<0.05, Figure 7j). With tetracycline and nalidixic acid the inhibition was 79.2% (Figure 7k) and 36.1% (Figure 7l), respectively. A representative control image of *B. pumilus* pre-formed biofilms is shown in Figure 7m. These were significantly disrupted (90.1%) with the antimicrobial protein (Figure 7n). With tetracycline and nalidixic acid the disruption was lesser with the values, 68.5% (Figure 7o) and 30.5% (Figure 7p), respectively.

### Scanning electron microscopy

**Figure 8:**
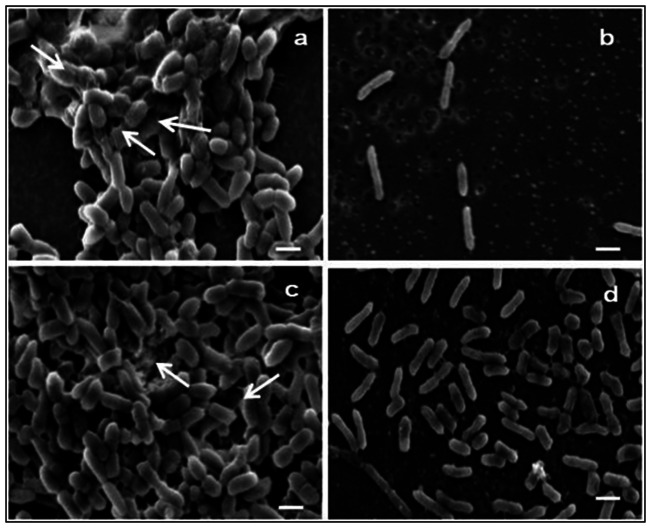
Representative SEM images of *P.* *aeruginosa* biofilms (a) control (b) co-incubated with protein BL-DZ1. Pre-formed biofilms (c) control (d) disruption by protein BL-DZ1. Bar represents 1 µm scale.

SEM images of *P. aeruginosa* control biofilms and those showing the effect of protein BL-DZ1 at MIC concentration were obtained. Figure 8a shows the observations made with a representative control sample. The antimicrobial protein BL-DZ1 was more effective in inhibiting biofilm formation during the co-incubation experiments (Figure 8b). A representative image of pre-formed biofilms (control) is shown in Figure 8c. Compared to the results obtained with the co-incubation experiments (Figure 8b), disruption of pre-established biofilms was to a lesser extent (Figure 8d). The presence of exo-polymeric substance (EPS) was evident in control samples (Figure 8a and c, white arrows), however this was not case in the test samples (Figure 8b and d).

## Discussion

Marine ecosystems are a potential source of novel antibiofilm compounds [5]. Marine microorganisms produce secondary metabolites in order to gain access to living space and to aid surface colonization. Epibiotic bacteria associated with different living organisms often produce novel compounds with commercial potential. In the present investigation, we studied the ability of a protein, BL-DZ1 derived from a tropical marine strain of *B. licheniformis* to inhibit biofilm formation and disperse pre-formed biofilms of *C. albicans*, *P. aeruginosa* and *B. pumilus*.

The marine strain of *B. licheniformis* D1 displayed antimicrobial activity that was first observed after 12 h of growth, and a maximum zone of inhibition was observed at 36 h (Figure 1a inset; white arrow). The bacterial growth (Figure 1a) and antimicrobial activity (Figure 1a inset; black arrow) decreased thereafter, due to auto-inhibition. A similar phenomenon has also been reported in case of the marine strain of *Pseudoalteromonas tunicata* isolated from the surface of a tunicate, *Ciona intestinalis* [28].

We observed that the antimicrobial activity in the cell-free supernatants of *B. licheniformis* was lost after treatment with proteolytic enzymes, suggesting that the antimicrobial compound could be a protein. This protein was purified to homogeneity (Figure 2 inset; lane c) by ultrafiltration and size-exclusion chromatography. Such techniques are effectively used in purifying antimicrobial proteins from other strains of *B. licheniformis* [29], [30]. A variety of proteins obtained from *B. licheniformis* are thermo- and pH- stable [29], [30], [31]. The purified protein (BL-DZ1) was stable at 75°C and over a wide range of pH, however the antimicrobial activity was lost after treatment with trypsin and proteinase K.

Characterization of the BL-DZ1 by MALDI-TOF MS/MS finger-printing technique suggested the molecular mass of 14 kDa (Figure 2; Figure S1). Tryptic digest fingerprint of BL-DZ1 matched with an NCBI entry for a hypothetical protein (encoded by the locus BL00275) from *B. licheniformis* ATCC 14580 [32], [Lundström S. (2012) Characterization of a *Bacillus licheniformis* gene cluster required for functional expression of a bacteriocin. Ph.D. thesis submitted to the Faculty of Science, University of Copenhagen]. Different strains of *B. licheniformis* produce a variety of antimicrobial compounds (Table 3), however the observed molecular mass of BL-DZ1 protein from *B. licheniformis* D1 was different from those reported earlier.

**Table 3:** Summary of antimicrobial proteins from *Bacillus licheniformis* strains.

*B. licheniformis*	Molecular mass (kDa)	Reference
*B. licheniformis* 26L10/3RA	1.4	[30]
*B. licheniformis*	30.7	[47]
*B. licheniformis* ZJU12	3.0	[39]
*B. licheniformis* BC98	1.035	[48]
*B. licheniformis* MKU3	1.5	[40]
*B. licheniformis* DSM13	3.02, 3.25	[26]
*B. licheniformis* IITRHR2	∼ 1.2	[49]
*B. licheniformis* EI-34-6	12.0, 30.0, 36.0	[43]
*B. licheniformis* D1	14.0	Present study

The protein (BL-DZ1) displayed antimicrobial activity against the fungus, *C. albicans* and the bacterial strains of *P. aeruginosa* and *B. pumilus*. The MIC values against *C. albicans* (1.60 µg/ml) and *P. aeruginosa* as well as *B. pumilus* (3.12 µg/ml) were significantly lower than other antimicrobial agents studied. We have previously reported a glycolipid biosurfactant obtained from a marine strain of *S. marcescens* that displayed antimicrobial activity at MIC concentrations of 12.5 and 25.0 µg/ml respectively against these fungal and bacterial strains [21]. In comparison with the aforementioned glycolipid, the antimicrobial protein (BL-DZ1) was more effective even at lower concentrations.

As per European Committee on Antimicrobial Susceptibility Testing (EUCAST, http://www.eucast.org) reports, the MIC values for fluconazole against *C. albicans* are in the range of 16–32 µg/ml. However the observed value in the present study was higher suggesting a possible resistance of this strain towards fluconazole. *P. aeruginosa* showed comparable MIC values (40 in current investigation and 32 µg/ml in an earlier report) with tetracycline [33]. With nalidixic acid however, the current values were higher (1250 µg/ml) than the previously reported MIC of 700 µg/ml [34]. Higher MIC values for some of the standard antibiotics highlight the need to study alternative antimicrobial agents.

The formation of microbial biofilms is often associated with a decrease in antimicrobial susceptibility; therefore inhibition of biofilm growth as well as dispersion of pre-formed biofilms is essential. Compounds derived from *Bacillus* sp. are reported to be effective against bacterial biofilms mainly during co-incubation. For example, a lipopeptide produced by *B. licheniformis* strain V9T14 inhibits biofilm formation of the human pathogens *Escherichia coli* and *Staphylococcus aureus* [35]. 4-phenylbutanoic acid obtained from a marine strain of *B. pumilus* is also reported to be effective in inhibiting bacterial biofilms [36]. Interestingly, the protein BL-DZ1 was able to inhibit both biofilm growth and disrupted pre-formed biofilms of all the test cultures (Figure 3a and 3c; Figure 4a and 4c; Figure 5a and 5c, Table 2). These results are in agreement with a report on the effectiveness of the antimicrobial substance (AMS) obtained from a strain of *B. licheniformis* (T6-5 from an oil-reservoir) against *B. pumilus* biofilms [37]. A variety of compounds affect bacterial as well as fungal biofilms. For example, chemically synthesized 2-aminoimidazole is known to inhibit a range of fungal and bacterial biofilms [38]. Similarly an enzymatically synthesized ester of lauroyl glucose displayed activity against both fungal and bacterial biofilms [27]. Most of the compounds derived from *B. licheniformis* are either antibacterial or antifungal in nature. However, some bacteriocins derived from this bacterium display both the activities [39], [40]. In agreement with these reports, BL-DZ1 was also effective against bacteria and the fungus. Bacteriocin-like proteins can affect microbial growth by a variety of mechanisms. They may display non-specific DNase activity, specific RNase activity, may induce pore formation or inhibit septum formation [41], [42]. The mechanism by which BL-DZ1 mediates antibiofilm activity needs to be investigated.

The type of surface influences microbial attachment and biofilm formation abilities. We observed that the protein is more effective against biofilms formed on glass surfaces than in polystyrene microtiter plates (Figure 3–7). There is an earlier report on the effective disruption of pre-established *P. aeruginosa* biofilms on glass surfaces by extracts from a marine strain of *B. pumilus* S6-15 [43]. Another compound [SN(3J6)] obtained from *Pseudoalteromonas* sp. has also been efficient in impairing *P. aeruginosa*, *Salmonella enterica* and *E. coli* biofilm formation on glass surfaces [44]. CLSM analysis of control and test biofilms revealed a large population of cells killed after treatment with the protein BL-DZ1 (Figure 6 and 7) due to loss of cell viability.

Biofilm exopolymeric substances (EPS) are important in biofilm establishment, architecture and may confer resistance towards antibiotics and biocides [45]. Amongst other mechanisms, removal of EPS makes the biofilm susceptible towards inhibitory compounds [46]. SEM analysis of *P. aeruginosa* biofilms revealed the presence of EPS (Figure 8a and 8c, white arrows). This EPS was removed after co-incubation of the protein with cells of *P. aeruginosa* and when applied onto the pre-formed biofilms (Figure 8b and 8d).

In conclusion, the protein BL-DZ1 isolated from a marine strain of *B. licheniformis* D1 effectively inhibited growth and dispersed pre-established biofilms. This study highlights the importance of the marine epibiotic bacteria as a potential source of antibiofilm compounds. Further research on such proteins would help in isolating a new class of antibiofilm compounds with broad spectrum activity. Further analysis on the mechanism of action of this protein in inhibiting biofilms is ongoing.

## Supporting Information

Figure S1 **Tryptic digest fingerprint of** ***B. licheniformis*** **antimicrobial protein after MALDI-TOF MS/MS analysis.**
(DOC)
