# Supplementary figures and images for: Disruption of Microbial Biofilms by an Extracellular Protein Isolated from Epibiotic Tropical Marine Strain of Bacillus licheniformis
Source: PLoS One. 2013 May 15;8(5):e64501. doi: 10.1371/journal.pone.0064501 (PMC3655075; doi:10.1371/journal.pone.0064501)

**
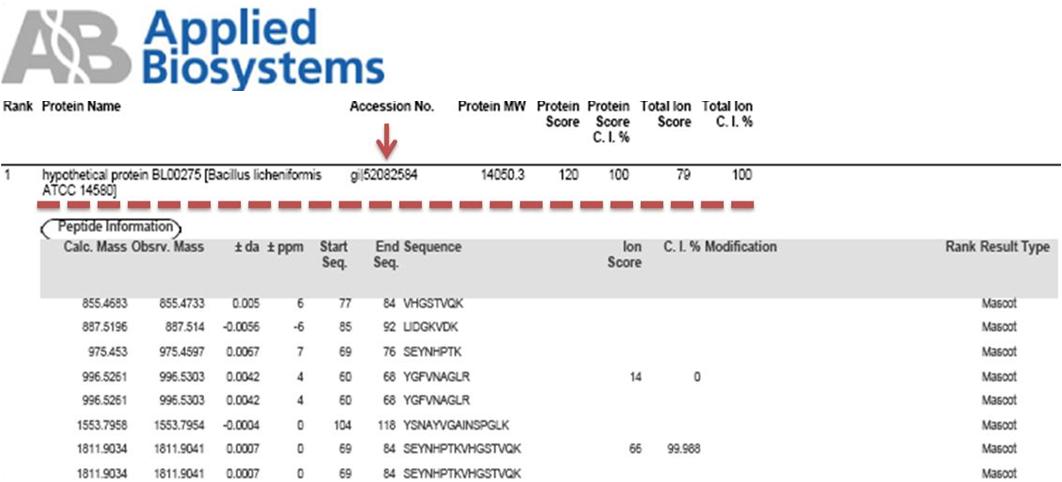
**

Supplement: Figure S1 — Tryptic digest fingerprint of B. licheniformis antimicrobial protein after MALDI-TOF MS/MS analysis. (DOC) [file pone.0064501.s001.doc]
